# Expression of Msx-1 is suppressed in bisphosphonate associated osteonecrosis related jaw tissue-etiopathology considerations respecting jaw developmental biology-related unique features

**DOI:** 10.1186/1479-5876-8-96

**Published:** 2010-10-13

**Authors:** Falk Wehrhan, Peter Hyckel, Jutta Ries, Phillip Stockmann, Emeka Nkenke, Karl A Schlegel, Friedrich W Neukam, Kerstin Amann

**Affiliations:** 1 Department of Oral and Maxillofacial Surgery University of Erlangen-Nuremberg Glueckstrasse 11, 91054 Erlangen, Germany; 2 Department of Plastic Surgery/St. Georg-hospital Eisenach University of Jena Erlanger Allee 101, 07747 Jena, Germany; 3 Institute of Pathology University of Erlangen-Nuremberg Universitaetsstrasse 22, 91054 Erlangen, Germany

## Abstract

**Background:**

Bone-destructive disease treatments include bisphosphonates and antibodies against the osteoclast differentiator, RANKL (aRANKL); however, osteonecrosis of the jaw (ONJ) is a frequent side-effect. Current models fail to explain the restriction of bisphosphonate (BP)-related and denosumab (anti-RANKL antibody)-related ONJ to jaws. Msx-1 is exclusively expressed in craniofacial structures and pivotal to cranial neural crest (CNC)-derived periodontal tissue remodeling. We hypothesised that Msx-1 expression might be impaired in bisphosphonate-related ONJ. The study aim was to elucidate Msx-1 and RANKL-associated signal transduction (BMP-2/4, RANKL) in ONJ-altered and healthy periodontal tissue.

**Methods:**

Twenty ONJ and twenty non-BP exposed periodontal samples were processed for RT-PCR and immunohistochemistry. An automated staining-based alkaline phosphatase-anti-alkaline phosphatase method was used to measure the stained cells:total cell-number ratio (labelling index, Bonferroni adjustment). Real-time RT-PCR was performed on ONJ-affected and healthy jaw periodontal samples (n = 20 each) to quantitatively compare Msx-1, BMP-2, RANKL, and GAPDH mRNA levels.

**Results:**

Semi-quantitative assessment of the ratio of stained cells showed decreased Msx-1 and RANKL and increased BMP-2/4 (all p < 0.05) expression in ONJ-adjacent periodontal tissue. ONJ tissue also exhibited decreased relative gene expression for Msx-1 (p < 0.03) and RANKL (p < 0.03) and increased BMP-2/4 expression (p < 0.02) compared to control.

**Conclusions:**

These results explain the sclerotic and osteopetrotic changes of periodontal tissue following BP application and substantiate clinical findings of BP-related impaired remodeling specific to periodontal tissue. RANKL suppression substantiated the clinical finding of impaired bone remodelling in BP- and aRANKL-induced ONJ-affected bone structures. Msx-1 suppression in ONJ-adjacent periodontal tissue suggested a bisphosphonate-related impairment in cellular differentiation that occurred exclusively jaw remodelling. Further research on developmental biology-related unique features of jaw bone structures will help to elucidate pathologies restricted to maxillofacial tissue.

## Introduction

Numerous attempts have targeted explaining the etiology of the restriction of amino-bisphosphonate (BP)-associated osteonecrosis of the jaw (BONJ) to the jaws, but an accepted model of formal pathology has been lacking [1,2]. Existing hypotheses have focused on accumulation of BP in the jaw or BP-specific tissue toxicity as a factor [3]. However, denusomab (humanized anti-RANKL antibody, Prolia, Amgen, USA) also has been demonstrated to cause osteonecrosis specifically of the jaw (ONJ) [4-6]. Thus, any hypothesized etiology of BONJ requires incorporation of these findings [1].

Potential factors to consider include the unique biological features of the alveolar bone of the jaw. Impairment of cranial neural crest (CNC)-specific RANKL-associated cell signaling as an underlying mechanism of ONJ is an attractive hypothesis because CNC-derived periodontal progenitor cells are involved in remodeling of both hard and soft jaw tissues [7-9]. Impairment of CNC cell plasticity affects remodeling of jaw bone and periodontal structures [7-9]. In addition, the transcription factor Msx-1 mediates the innate cellular plasticity of CNC and is expressed exclusively in CNC-derived bone and bone progenitor structures including oral periost and periodontal ligamentum (PDL) throughout adolescence [10,11]. Within the jaw, Msx-1 is expressed with the highest concentration in the PDL [9,11-13] and is co-expressed with RANKL on CNC-derived osteoblast and chondroblast progenitors [14-16]. Because of the restriction of Msx-1 to the adult jaw and its co-expression with RANKL, a BP- and denusomab-related loss of RANKL and Msx-1 expression might explain the BP- and denosumab-related impairment of hard and soft tissue remodeling that is restricted to the jaw bone in ONJ [4,14]. Thus, the aim of this study was to compare Msx-1, BMP-2/4, and RANKL expression at the protein and mRNA levels in samples of BONJ-related oral mucoperiosteal tissue compared to healthy oral periodontal tissue to test the hypothesized impairment of jaw-specific Msx-1-RANKL-associated cell signaling in periodontal progenitor cells.

## Materials and methods

### Patients and Material Harvesting

This study included oral mucoperiosteal specimens from 40 patients. Of these, 20 were from periodontal soft tissue adjacent to clinically and histologically confirmed BONJ of 20 consecutively treated patients undergoing radical sequestrotomy, taken as part of the tissue samples provided for routine histopathological diagnostics. The study was approved by the ethical committee of the University of Erlangen-Nuremberg. All patients gave their informed consent to participation. Additional criteria for specimen inclusion were intravenous application of either pamidronate or zoledronate for at least 12 months and clinical evidence of an exposed jaw bone for at least 8 weeks. Any former radiotherapy was excluded. Details about patient data, surgical treatment, and the follow-up period were previously documented [17]. Controls were 20 alveolar mucoperiosteal specimens, harvested during intraoral surgery in patients negative for BP history and presenting no clinical signs of intraoral inflammatory processes or periodontitis. The 40 specimens measured on average 5 × 3 × 3 mm and were immediately separated into two equal parts. One part was immediately flash frozen at -80°C in liquid nitrogen. Mature bone pieces were detached from the other part, and the periodontal soft tissue was immersed in RNA-preserving reagent (RNALater, Qiagen, Hilden, Germany) for 24 h at 4°C and then frozen and stored at -80°C.

### Immunohistochemical Staining

Tissue samples were processed for immunohistochemistry as previously described[18]. Antibodies and dilutions were as follows: Msx-1, polyclonal rabbit-IgG anti-human Msx-1 antibody (anti-Msx-1; M0944-100G, Sigma-Aldrich, Taufkirchen, Germany; dilution 1:100); BMP-2/4, polyclonal rabbit-IgG (anti-human BMP-2/4, sc-9003, Santa Cruz Biotechnology, Santa Cruz, CA, USA; dilution: 1:100); and RANKL, polyclonal goat-anti-human RANKL antibody (sc-7628, Santa Cruz, dilution 1:100). Secondary antibody was used according to the staining kit [biotinylated polyclonal, goat-anti-rabbit IgG (Msx-1, BMP-2/4) and rabbit-anti-goat (RANKL) (E 0466, DAKO, dilution 1:100)]. Visualization was performed using Fast Red solution, and localized by biotin-associated activation of the staining kit (ChemMate-Kit, Dako) followed by incubation in hematoxylin for nuclear counterstaining. Two consecutive tissue samples were processed per immunohistochemical staining, one for experimental staining and the other as a negative control (replacement of primary antibody incubation with incubation with istotype-IgG of the primary antibody). A known positive staining sample was also included in each series as a positive control.

### Semiquantitative Immunohistochemical Analysis

Sections were examined qualitatively under a bright-field microscope (Axioskop, Zeiss, Jena, Germany) at 100-400× magnification for number and localization of stained osteoblast progenitors and fibroblasts. In healthy periodontal samples, subepithelial tissue was observed, including connective, submucous, and periosteal structures. Mature bone tissue, including osteocytes, was excluded from any analysis. In BONJ samples, soft tissue adjacent to the necrotic zone was identified, and three visual fields per section for each sample were digitized at 200× magnification using a CCD camera (Axiocam 5, Zeiss, Jena, Germany) and the program AxioVision (AxioVison, Zeiss, Jena, Germany). For this purpose, randomized systematic subsampling was performed as previously described [18]. Semiquantitative analysis of cytoplasmic expression of Msx-1, BMP-2/4, and RANKL was performed by determining the labeling index as the ratio of positively stained cells to the total number of cells per visual field.

### Quantitative mRNA Analysis and Real-time Reverse Transcriptase Polymerase Chain Reaction (RTqPCR)

Frozen tissues were agitated (Mixer Mill, Qiagen, Hilden, Germany) in lysis buffer (RNeasy Kit, Qiagen, Hilden, Germany), and whole RNA from tissues was extracted using the RNeasy Kit according to the manufacturer's protocol. Quantitative measurement of mRNA in each probe was performed using a commercial microfluid Lab-on-a-Chip technology (Agilent RNA 6000 Pico Kit and the Agilent 2100 Bioanalyzer, Agilent, Waldbronn, Germany). The cDNAs from total RNA were synthesized using the High Capacity cDNA Archive Kit (Cat. 4322171; Applied Biosystems, Foster City, CA, USA) according to the manufacturer's protocol. Real-time RT qPCR analyses were done using QuantiTect Primer Assay (200) [Hs_BMP2_1_SGQuantiTect Primer Assay (200) (Cat. GT00012544) for BMP-2; Hs_MSX1_SG QuantiTect Primer Assay (200) (Cat. GT00224350) for Msx-1; and Hs_TNFSF11_va.1_SG QuantiTect Primer Assay (200) (Cat. QT01011381) for RANKL]. For normalization, GAPDH was used [Hs_GAPDH_1_SG QuantiTect Primer Assay (200) (Cat. QT00079247), Qiagen)]. The QuantiTect TM SYBR Green PCR kit (Cat. 204143; Qiagen) was used for PCR amplification. The relative quantification of mRNA was performed with the ABI Prism 7300 Sequence Detection System (Applied Biosystems). In total, 40 ng of cDNA was used for each PCR reaction in a total volume of 25 μl. Each PCR run included a 15-min activation time at 95°C, followed by a three-step cycle: denaturing at 94°C for 15 s, annealing at 55°C for 30 s, and extension at 72°C for 34 s. Formation during PCR of undesired side products that contribute to fluorescence was assessed by melting curve analysis after PCR. Msx-1, BMP-2, and RANKL mRNA quantities were analyzed in duplicate, normalized against GAPDH as an internal control, and expressed in relation to mRNA isolated from healthy periodontal tissue as a calibrator. Relative gene expression was determined using the ΔΔCt method. RNA isolated from healthy oral periodontal tissue (pool of 20 patients) was used as controls.

### Statistical Analysis

To analyze the immunohistochemical cytoplasmic staining and the spatial pattern of expression, the labeling index of positively stained cells per visual field was assessed. Comparing the relative gene expression, addressed by the real-time RT-PCR, the median gene expression for Msx-1, BMP-2, and RANKL in the pool of healthy oral mucoperiosteum was set as 1. Gene expression in both groups was stated as relative expression compared to healthy mucoperiosteal expression. Multiple measurements per group of investigation were aggregated prior to analysis. Descriptive analysis of labeling index and relative gene expression data were performed using the median (ME) and the interquartile range (IQR). Graphical representations use diagrams representing ME, IQR, minimum, and maximum. Confirmatory comparisons were made between treatment and control groups using generalized estimating equations with "treatment modality" and "subject id" as independent factors for appropriate analysis of repeated measurements per individual. Multiple p values were adjusted according to Bonferroni by multiplying each p value obtained by the number of confirmatory tests performed (n = 10). Two-sided adjusted p values of p ≤ 0.05 were considered to be significant. All calculations were made using SPSS 18.0 for Windows (SPSS Inc, Chicago, IL, USA).

## Results

### Immunohistochemistry

**Figure 1:**
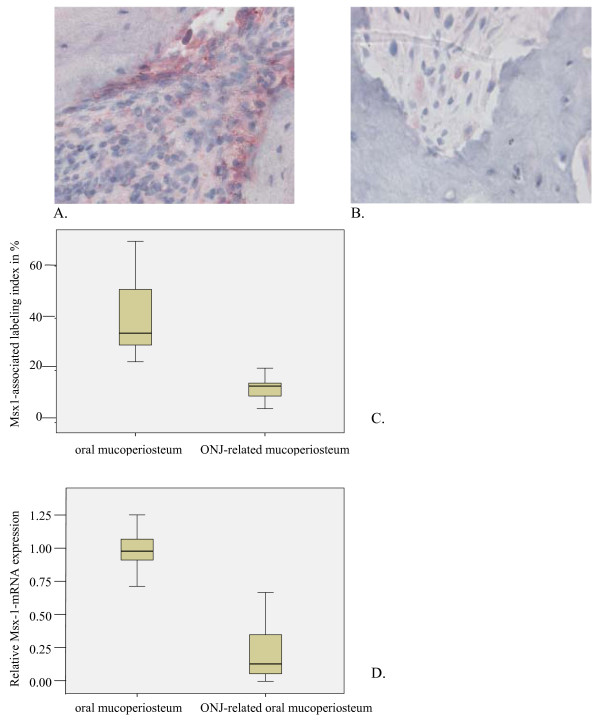
**Msx-1 expression was reduced in ONJ-related periodontal tissue**. (a) The Msx-1 staining was accentuated in periosteal cells, attached to the mineralized bone matrix. The bone trabeculae interconnecting fibrous tissue presented nuclear and cytoplasmic Msx-1 staining. (b) In the BONJ group, staining of periosteal cells was rare, and cytoplasmic staining was decreased, as was the cellular density of Msx-1-expressing fibroblasts in the fibrous and inflammatory tissue surrounding the bone matrix. (c) Relative cellular expression (labeling index) for Msx-1 was significantly reduced (Controls-ME: 34.29, IQR 24.0 vs. BONJ-ME: 14.03, IQR: 6.0; p < 0.05) in ONJ-related oral mucoperiosteum. (d) Relative gene expression for Msx-1 was suppressed 6.8-fold at the mRNA level in ONJ-related periosteum samples (Controls-ME: 1.00, IQR 0.25 vs. BONJ-ME: 0.15, IQR: 0.31; p < 0.03). Horizontal bars indicate median (ME), and error bars indicate interquartile range (IQR).

**Figure 2:**
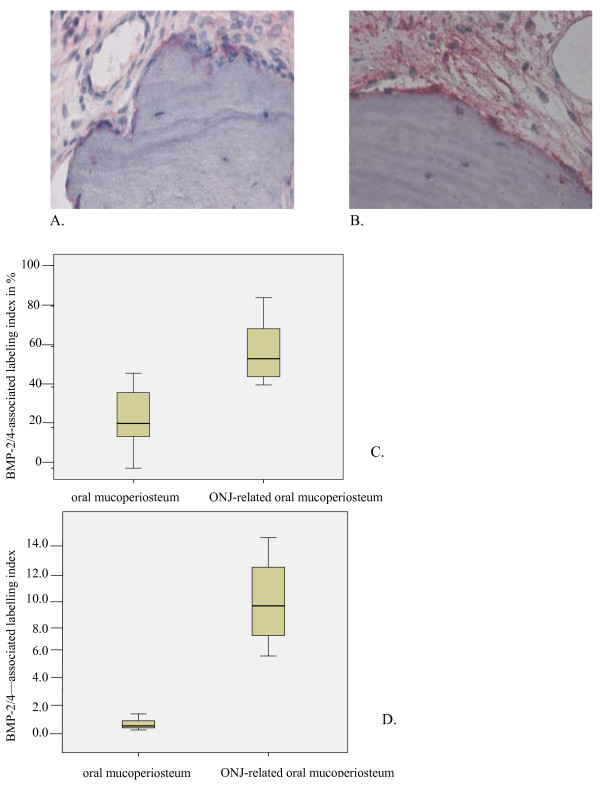
**BMP-2/4 expression was increased at the protein and mRNA levels in BP-altered oral mucoperiosteum**. (a) Rarely, there was pronounced BMP-2/4 staining in healthy jaw periosteum. (b) BMP-2/4-expressing osteocytes showed higher cellular density in the BONJ group. (c) The labeling index of BMP-2/4-expressing osteoblasts and osteocytes was significantly increased compared to control (Controls-ME: 22.06, IQR 25.0 vs. BONJ-ME: 53.97, IQR: 25.0; p < 0.05). (d) Relative BMP-2 gene expression at the mRNA level was elevated 8.9-fold in ONJ samples (Controls-ME: 1.14, IQR 1.07 vs. BONJ-ME: 8.9, IQR: 6.1; p < 0.02) related to healthy samples. Horizontal bars indicate median (ME), and error bars indicate interquartile range (IQR).

**Figure 3:**
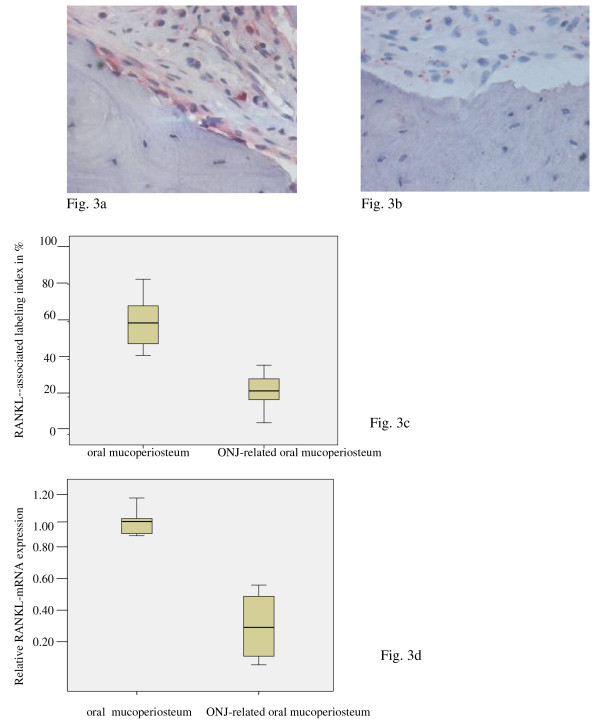
**RANKL was suppressed in ONJ-adjacent soft tissue**. (a, b) Spatial distribution of RANKL-expressing cells in the soft tissue areas of BONJ samples (b) was non-homogeneous compared to normal jaw periodontal samples (a). A local high concentration of RANKL-expressing multinucleated cells was detected only at zones of tissue resorption in BONJ samples. (c) The relative cellular expression (labeling index) of RANKL-positive cells was significantly lower in ONJ samples (Controls-ME: 59.38, IQR 21.0 vs. BONJ-ME: 23.25, IQR: 12.0; p < 0.05). (d) A 2.94-fold suppression of RANKL mRNA was detected in ONJ-related bone samples (Controls-ME: 1.00, IQR 0.13 vs. BONJ-ME: 0.34, IQR: 0.44; p < 0.03). Horizontal bars indicate median (ME), and error bars indicate interquartile range (IQR).

All examined BONJ samples had multinucleated cells and a thickened epithelial layer above necrotic tissueareas between vital zones (Figures 1b, 2b, 3b). Observation consistently showed necrotic lesions of partial confluency. Empty osteocyte lacunae were detected. The mucoperiosteal soft tissue presented variable thickness including inflammatory infiltrates within the connective tissue layers. Capillaries were seen in BONJ-related mucoperiosteal specimens and healthy jaw connective tissue.

In control jaw periodontal tissue, Msx-1 expression was localized in the nucleus and cytoplasm of osteoblasts, fibroblasts, and progenitors within the connective tissue layer (Figure 1a). In the BONJ-related tissue, a reduced cellular density of Msx-1 expressing osteoblasts, fibroblasts, and progenitor cells was noted (Figure 1b). BMP-2/4 expression was found in osteoblast progenitors of adjacent periosteal tissue in both healthy jaw bone (Figure 2a) and the BONJ samples (Figure 2b).

RANKL expression was present throughout the soft tissue in normal jaw samples (Figure 3a), including periosteal and subepithelial tissue; however, in BONJ samples, RANKL expression was present sparsely in the endosteal and periosteal tissue at the site of bone resorption (Figure 3b).

The labeling index of Msx-1-expressing (Figure 1c) and RANKL-expressing (Figure 3c) cells was significantly diminished compared to normal bone. The labeling index of BMP-2/4-expressing osteoblasts and osteocytes (Figure 2c) was significantly increased compared to control.

### PCR

The patterns for mRNA expression reflected those for protein expression. Msx-1 mRNA levels were significantly suppressed 6.8-fold in BONJ samples compared to control periodontal tissue (Figure 1d). BMP-2/4 mRNA expression was significantly higher by about 8.9-fold in BONJ tissue than in normal jaw mucoperiosteal tissue (Figure 2d), while RANKL mRNA expression was significantly suppressed 2.9-fold in BONJ samples relative to control (Figure 3d).

## Discussion

This study identified a significantly diminished expression of Msx-1, a cellular plasticity and proliferation-mediating transcription factor, in BONJ-affected jaw periodontal tissue at the protein and mRNA levels. Significantly elevated expression of BMP-2/4 in the BONJ-related periodontal and periosteal tissue revealed an increased osseous differentiation stimulation in progenitors of osteoblastic lineage in BP-compromised jaw mucoperiosteal tissue. As with Msx-1 expression, RANKL expression in the jaw bone overlying mucoperiosteal tissue was significantly reduced, suggesting suppressed osteoclast activation by osteoblasts [19].

BP-related Msx-1 loss in the PDL can explain the sclerotic, periapical hypermineralized thin lines around dental roots of BP-altered PDL tissue, which is known for having the highest endogenous Msx-1 expression in the jaw [9,12,13,20]. In addition, Msx-1 is critically involved in cellular plasticity and differentiation. Within the PDL, a balanced progenitor cell differentiation towards fibrous soft tissue takes place between dental and bone hard tissue. The clinical observation of sclerotic remodeling of the PDL is substantiated by the experimental finding of BP-induced osteogenetic cell recruitment and trans-differentiation of progenitor cells within the PDL [21]. Because Msx-1 has been reported to prevent terminal differentiation and to stimulate proliferation of progenitors, loss of Msx-1 in the presence of BMP-2 is likely to be associated with poor cell proliferation and also with overwhelming mineralization in periodontal tissue [22,23].

The significantly increased expression of BMP-2/4 identified here at the cellular and mRNA levels in BONJ-affected jaw periosteum is consistent with the clinical and radiologic observation of the osteopetrotic aspect of ONJ-related jaw bone: BMP-2/4 is an essential osteoinductive factor and induces terminal osseous differentiation through DLX5 signaling in the absence of Msx-1 [24]; [25]. Increased terminal osseus differentiation and reduced proliferation of progenitor cells within the periodontal tissue might explain sclerosis and osteopetrosis of the alveolar bone and the reduced periodontal soft tissue proliferation. The immunohistochemical and molecular results in this study are consistent with those found in osteopetrotic bone [26], and BONJ has been described as local osteopetrosis [24,27].

The finding of BP-related RANKL suppression in periodontal progenitor cells *in vivo *is described here for the first time and indicates the relevance of BP effects on cellular differentiation in explaining the etiology of BONJ. The significantly reduced expression of RANKL in ONJ-adjacent periodontal tissue at the protein and mRNA levels demonstrates the effect of BP action on soft-tissue remodeling. Suppression of RANKL has been described as the main action of BP, preventing osteoclast activation and bone resorption in malignancies and osteoporosis [28-31]. This suggestion finds strong support from clinical findings of ONJ onset following application of the anti-RANKL denosumab without any BP involvement [4,6]. The concerted regulation of RANKL and Msx-1 identified here connects jaw-specific and common bone remodeling mechanisms, but the details remain to be elucidated at the cellular and subcellular levels.

## Conclusion

These findings help to explain some of the molecular underpinnings of the restriction of BONJ to the jaw bone. Jaw restricted osteopetrosis implicated in BONJ can be explained by loss of Msx-1. Msx-1, known to be a key regulator of cellular plasticity and constitutively expressed in CNC-derived jaw hard and soft tissue progenitor cells, could be of relevance in jaw-restricted diseases associated with impaired bone and soft tissue remodeling [32-34]. Addressing the Msx-1-RANKL-associated signaling could help to elucidate mechanisms of CNC-related jaw bone and periodontal-tissue-specific homeostasis [7-9]. In agreement with leading international experts in the field of ONJ, we found that targeting the unique features of the jaw bone is a promising approach to elucidating the underlying pathologic mechanisms of ONJ [35]. Of note, BP and aRANKL had differential impacts on proliferation, vascularisation, and surface marker expression [36,37]. This suggests that BP and aRANKL effects on Msx- and RANKL-related interactions in CNC- and MsC-derived osteoblasts, osteoclasts, and bone structures should be investigated in more detail in the future.

## Competing interests

There are no competing interests of the authors to be declared.

This study was funded by the ELAN-Fonds of the University of Erlangen-Nuremberg, Germany.

## Authors' contributions

FW was responsible for the application of grant support (ELAN-Fonds, university of Erlangen), the conduction of study, built the hypothesis, established and conducted the methods and analytic procedures and wrote the manuscript. PH built the hypothesis and did the interpretation of the data. JR established the m-RNA analysis and RT-PCR and wrote the manuscript, section RT-PCR. PS and KS did the immunohistochemistry analysis.

FN interpreted the data and wrote the manuscript, section discussion. EN interpreted the data and conducted the study by harvesting samples. KA established immunohistochemistry, analysed the tissue samples, interpreted the data and was responsible for the histopatholgical analysis of ONJ- and control tissue samples. All authors read and approved the final manuscript.
